# Optimization of HER3 expression imaging using affibody molecules: Influence of chelator for labeling with indium-111

**DOI:** 10.1038/s41598-018-36827-w

**Published:** 2019-01-24

**Authors:** Sara S. Rinne, Charles Dahlsson Leitao, Bogdan Mitran, Tarek Z. Bass, Ken G. Andersson, Vladimir Tolmachev, Stefan Ståhl, John Löfblom, Anna Orlova

**Affiliations:** 1 Department of Medicinal Chemistry, Uppsala University, Uppsala, Sweden; 2 Department of Protein Science, School of Engineering Sciences in Chemistry, Biotechnology and Health, KTH Royal Institute of Technology, Stockholm, Sweden; 3 Department of Immunology, Genetics and Pathology, Uppsala University, Uppsala, Sweden; 4 Science for Life Laboratory, Uppsala University, Uppsala, Sweden

## Abstract

Radionuclide molecular imaging of human epidermal growth factor receptor 3 (HER3) expression using affibody molecules could be used for patient stratification for HER3-targeted cancer therapeutics. We hypothesized that the properties of HER3-targeting affibody molecules might be improved through modification of the radiometal-chelator complex. Macrocyclic chelators NOTA (1,4,7-triazacyclononane-N,N′,N′′-triacetic acid), NODAGA (1-(1,3-carboxypropyl)-4,7-carboxymethyl-1,4,7-triazacyclononane), DOTA (1,4,7,10-tetraazacyclododecane-1,4,7,10-tetraacetic acid), and DOTAGA (1,4,7,10-tetraazacyclododececane,1-(glutaric acid)−4,7,10-triacetic acid) were conjugated to the C-terminus of anti-HER3 affibody molecule Z_08698_ and conjugates were labeled with indium-111. All conjugates bound specifically and with picomolar affinity to HER3 *in vitro*. In mice bearing HER3-expressing xenografts, no significant difference in tumor uptake between the conjugates was observed. Presence of the negatively charged ^111^In-DOTAGA-complex resulted in the lowest hepatic uptake and the highest tumor-to-liver ratio. In conclusion, the choice of chelator influences the biodistribution of indium-111 labeled anti-HER3 affibody molecules. Hepatic uptake of anti-HER3 affibody molecules could be reduced by the increase of negative charge of the radiometal-chelator complex on the C-terminus without significantly influencing the tumor uptake.

## Introduction

During recent years, targeting of the human epidermal growth factor receptor type 3 (HER3) has become an important and promising approach for anti-cancer therapy. HER3 is one of the most potent activators of the PI3K/Akt signaling pathway^1,2^ and is known to be central for the maintenance and regulation of HER-signaling^2^. In cancer, HER3 can stimulate the oncogenic activity of epidermal growth factor receptor (EGFR) and human epidermal growth factor receptor 2 (HER2) and the resistance to HER-targeted cancer therapies^3,4^. Current data suggest that overexpression or aberrant expression of HER3 is associated with different types of cancer^4^. For example, overexpression of HER3 is present in breast cancers^4,5^ and its co-expression with HER2 is reported to promote cell survival, proliferation, progression and metastasis of the disease^6,7^. Furthermore, elevated levels of HER3 have been linked to development and progression of hormone refractory prostate cancer^4^, tumorgenesis of gastric cancer^8^ and reduced survival in both of these diseases^8,9^.

A number of therapeutic agents against HER3 are currently being developed and are in different stages of clinical evaluation^10,11^. Due to the rapid progress in therapy development and existing therapy options, it is important to have dependable methods for identification of patients who would benefit from this type of specific treatment. However, conventional methods for identification of target expression are complicated: HER3 is not shedded at a sufficiently high amount to be a reliable biomarker in blood^12^ and biopsies are invasive procedures associated with risk of false negative results due to tumor heterogeneity. On the opposite, radionuclide molecular imaging modalities, such as positron emission tomography (PET) and single photon emission tomography (SPECT), are promising alternatives. Such molecular imaging modalities are non-invasive and repeatable methods for detection of target expression in primary tumors and metastases as well as for monitoring of therapy response^13^.

Imaging of HER3 has three major challenges. Firstly, overexpression is only observed in certain subset of tumors and often in response to therapy^14^. Secondly, the level of overexpression is relatively low, as it commonly does not exceed 50.000 receptors/cells^15^. Thirdly, substantial endogenous expression, particularly in liver, decreases imaging contrast and complicates image interpretation.

Affibody molecules are small-sized (6-7 kDa) three-helical scaffold proteins^16^. Rapid blood clearance and good tumor penetration provide high imaging contrast shortly after injection, making affibody molecules promising agents for detection of molecular target expression and patient stratification, which has been demonstrated in clinical trials for HER2-tageting affibody molecules in breast cancer patients^17,18^. The first radiolabeled HER3-targeting affibody molecules were reported in 2014 by Orlova *et al*. and demonstrated that the technetium-99m labeled anti-HER3 affibody molecule is feasible for SPECT-imaging of HER3-expressing malignant tumors^19^. Other studies further reported encouraging results for anti-HER3 affibody molecules radiolabeled with indium-111, gallium-68 and fluorine-18^20–22^. Affibody molecules provide not only reasonable imaging contrast shortly after injection, but also enable differentiation between xenografts with high and low levels of HER3 expression^21^. Despite promising results, high activity uptake by endogenously expressed receptors in liver, stomach, small intestine, lung and salivary glands remains a challenge for the detection of HER3 overexpression *in vivo*.

Selection of a chelator providing a thermodynamically stable and kinetically inert complex with a radionuclide is of paramount importance for development of an imaging probe. However, the molecular design and radiolabeling approach might have also noticeable effects on the biodistribution characteristics of the molecule, which in return can influence the sensitivity and specificity of the imaging agents^23–25^. It has been previously demonstrated with anti-HER2 affibody molecules that the interplay of the radiometal and the chelator can affect blood clearance as well as renal and hepatic uptake of affibody molecules due to variation in structure and local distribution of charge^26–28^. For example, HER2-targeting affibody molecules bearing a negatively charged radiometal-chelator complex at the N-terminus, showed significantly faster blood clearance and lower uptake in liver than the variants with complexes of positive or neutral charge^28^. As a result, modifications in design and labeling of affibody molecules provide an important tool for optimization of image sensitivity and specificity.

The effect of local charge on hepatic uptake is of special interest in the context of HER3 targeting. Liver has high levels of HER3-mediated uptake due to high endogenous receptor expression. Because liver is a prominent location for metastases in a number of cancers, elevated tracer accumulation in liver would potentially limit clinical utility of the imaging agent.

Moreover, off-target interactions, possibly related to the local charge on the targeting molecules, are considered to be a noticeable contributor to the hepatic uptake of anti-HER3 affibody molecules^29^. Previous studies using the anti-HER3 affibody molecule (HE)_3_-Z_08698_-NOTA demonstrated that labeling with radiocobalt provided the lowest hepatic uptake, compared to its indium-111 and gallium-68 labeled counterparts^20,21,29^. At 24 h pi, hepatic uptake of ^57^Co-(HE)_3_-Z_08698_-NOTA was lower than the uptake in the xenografts^29^. Cobalt, in contrast to indium and gallium, forms complexes in a divalent state. Hence, the use of radiocobalt reduces the charge of the radiometal-NOTA-complex from +1 for indium and gallium to zero. It was speculated that by exclusion of the positive charge unspecific uptake of anti-HER3 affibody molecules in liver might be reduced^29^.

**Figure 1:**
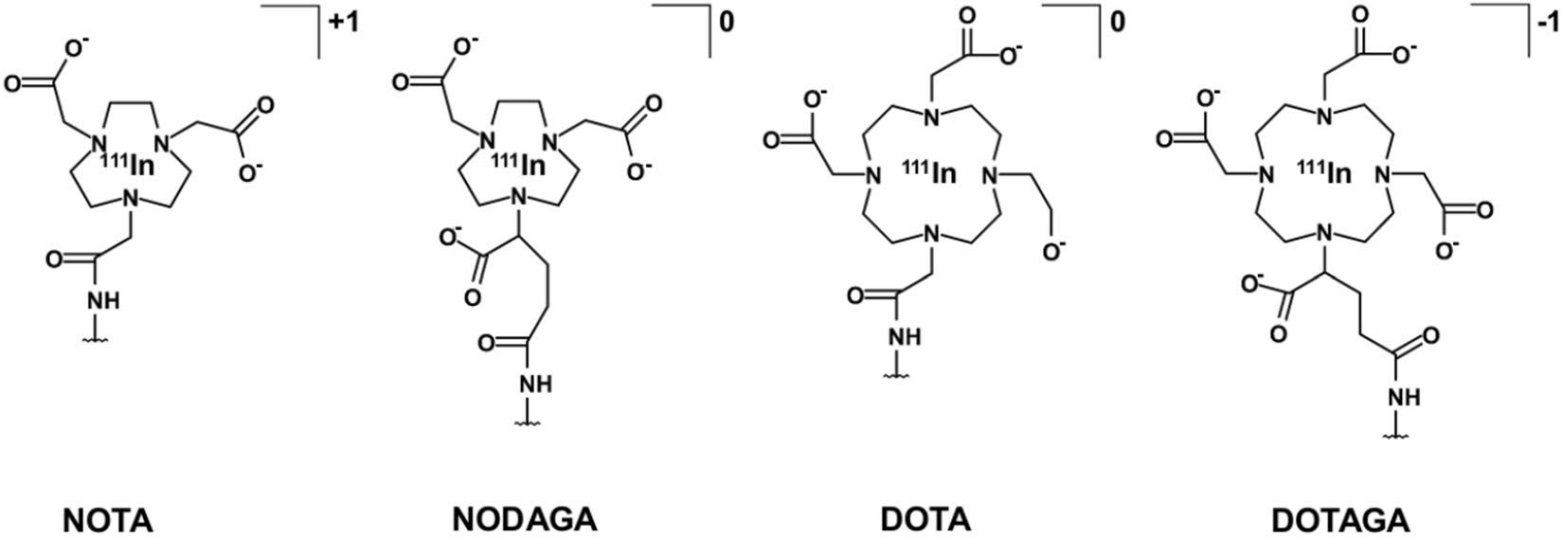
Structural overview of macrocyclic maleimide chelators 1,4,7-triazacyclononane-N,N′,N′′-triacetic acid (NOTA), 1-(1,3-carboxypropyl)-4,7-carboxymethyl-1,4,7-triazacyclononane (NODAGA), 1,4,7,10-tetraazacyclododecane-1,4,7,10-tetraacetic acid (DOTA) and 1,4,7,10-tetraazacyclododececane,1-(glutaric acid)-4,7,10-triacetic acid (DOTAGA) and charge of the complexes formed with indium-111 when conjugated to Z_08698_.

We hypothesize that the uptake in liver can be further decreased by increasing the negative charge through modification of the radiometal/chelator complex on the C-terminus of anti-HER3 affibody molecules. We thus investigated the influence of different indium-111/chelator complexes on the biodistribution of the anti-HER3 affibody molecule Z_08698_. Maleimide derivatives of four commonly used and commercially available macrocyclic chelators (X = NOTA, NODAGA, DOTA, DOTAGA; Fig. 1) were conjugated to Z_08698_ (hereafter denoted as Z_08698_-X) via a unique C-terminal cysteine. In contrast to earlier studies, no (HE)_3_-tag was conjugated to the N-terminus of Z_08698_ to investigate the pure influence of the C-terminal charge. *In vitro* and *in vivo* properties of all radio-conjugates were investigated in order to select the conjugate providing the best imaging properties for SPECT-imaging of HER3 expression *in vivo*.

## Results

### Production, conjugation, purification and characterization

The HER3-binding affibody Z_08698_ was recombinantly produced in *E*. *coli*, recovered with cation exchange chromatography, coupled to maleimide derivatives of NOTA, NODAGA, DOTA and DOTAGA and subjected to reverse-phase high performance liquid chromatography (RP-HPLC) as a final step for remnant chelator removal and separation from unconjugated protein. The purity, as determined by RP-HPLC, exceeded 98% for all four conjugates (Supplementary Fig. S1).

Molecular mass determination with electrospray ionization mass spectrometry (ESI-MS) confirmed the identity of the conjugated proteins and the observed masses were in good agreement with the theoretical (Table 1 and Supplementary Figure S2). Notably, the proteins conjugated to NOTA and NODAGA exhibited additional peaks which is likely the result of chelated metal contaminants.

**Table 1:** The experimental molecular masses, affinities and melting temperatures of the conjugates.

	Mw (Da)	K_D_ (pM, mean ± SD)* 1500 RU	K_D_ (pM, mean ± SD)** 2500 RU	T_m_ (°C)
Z_08698_-DOTA	7320.6 (7320.2)	12 ± 2.6	13 ± 2.9	64.5
Z_08698_-DOTAGA	7392.7 (7392.3)	15 ± 0.4	16 ± 0.8	64.0
Z_08698_-NOTA	7219.6 (7219.1)	40 ± 1.5	45 ± 6.7	63.8
Z_08698_-NODAGA	7291.5 (7291.2)	11 ± 0.6	13 ± 0.05	64.3

The theoretical molecular mass is in parenthesis.

*His-HER3 immobilized surface with 1500 RU (resonance units).

**mFc-HER3 immobilized surface with 2500 RU.

The alpha-helical content, thermal stability and refolding of the conjugates were investigated with circular dichroism spectroscopy. The thermal denaturation curves for the constructs are shown in Supplementary Fig. S3A and the associated melting temperatures are presented in Table 1. Following thermal denaturation, complete refolding of all four conjugates was evident from comparison of spectra obtained at 20 °C before and after denaturation (Supplementary Figure S3B). Kinetic data acquired from surface plasmon resonance (SPR) analysis are presented in Table 1. K_D_ values refer to the monovalent affinity for human HER3 according to a Langmuir 1:1 model. Representative sensorgrams with fitted curves for each conjugate are shown in Supplementary Fig. S4.

### Labeling and Stability

Results for radiolabeling of Z_08698_-X with indium-111 are shown in Table 2. For radiolabeling, the conjugates were dissolved in ammonium acetate (0.2 M, pH 5.5) and incubated with 20 MBq indium-chloride for 40 minutes at 85 °C. ^111^In-Z_08698_-NODAGA and ^111^In-Z_08698_-DOTAGA were successfully labeled with almost quantitative yields. Radiochemical yields for ^111^In-Z_08698_-NOTA and ^111^In-Z_08698_-DOTA were lower. Purification with NAP5 size-exclusion columns resulted in high radiochemical purity. Despite differences in labeling efficiency, all conjugates demonstrated high metal/chelator complex stability when challenged in human serum at physiological temperature (Table 2).

**Table 2:** Labeling and stability. Average labeling yield (n = 3–5) and stability in human serum.

	^111^In-Z_08698_-NOTA	^111^In-Z_08698_-NODAGA	^111^In-Z_08698_-DOTA	^111^In-Z_08698_-DOTAGA
Labeling Yield (%)	45 ± 13	96 ± 1	72 ± 4	97 ± 2
Purity after NAP5 purification	99 ± 2	99.9 ± 0.1	97.3 ± 0.9	100
% Release in Serum (24 h)	3 ± 1	0	4.8 ± 0.7	2 ± 2

Stability was evaluated by incubation in human serum for 24 h at 37 °C and is presented as % released activity.

### *In vitro* studies

**Figure 2:**
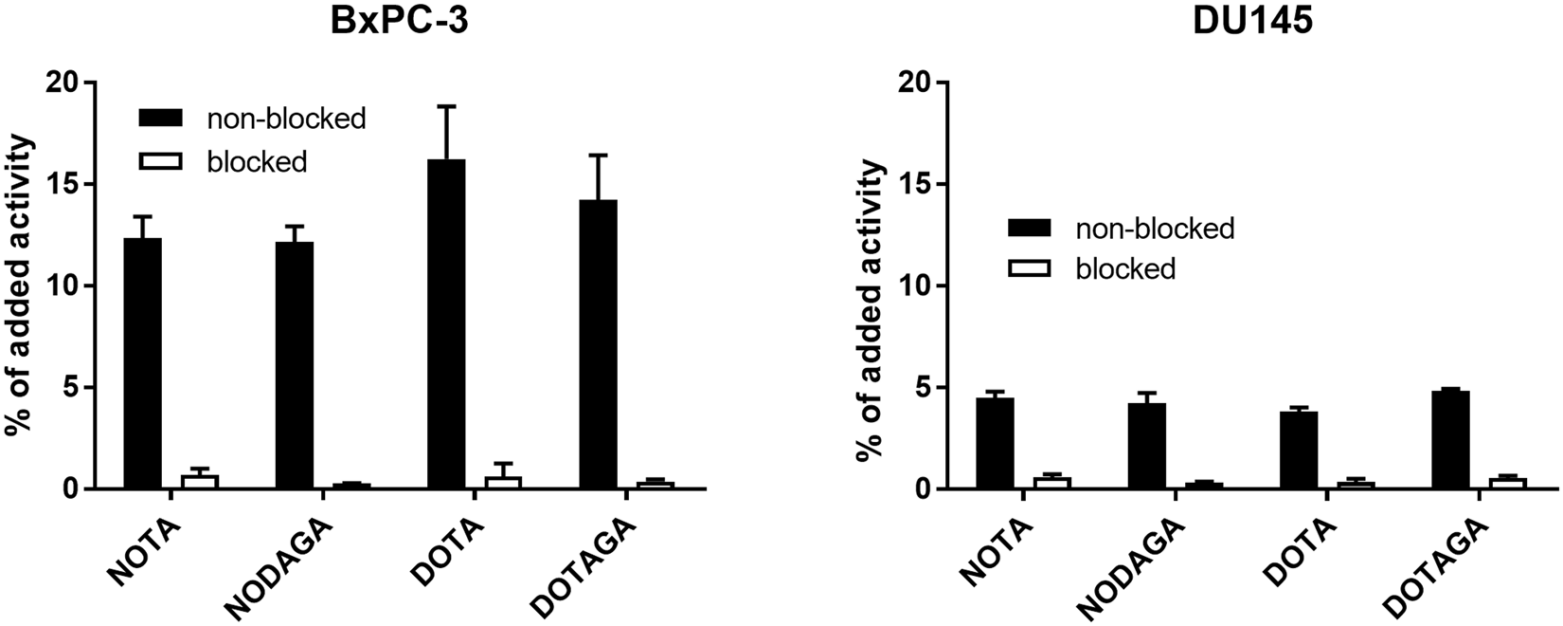
Binding specificity of ^111^In-Z_08698_-X on BxPC-3 and DU145 cells. HER3 receptors in the blocked group were pre-saturated with excess of unlabeled Z_08698_. Data are presented as an average ± standard deviation from three cell culture dishes.

*In vitro* studies were performed using BxPC-3 (pancreatic cancer) and DU145 (prostate cancer) cells. To demonstrate binding specificity, HER3 receptors were pre-saturated with 500-fold molar excess of a non-labeled HER3-tageting affibody molecule before incubation with the radiolabeled conjugates. Pre-saturation resulted in significantly decreased uptake of the radiolabeled conjugates. Hence, all tested conjugates retained receptor-specific binding after labeling as shown in Fig. 2. Overall, the uptake of all conjugates was lower for DU145 cells compared to BxPC-3 cells.

**Figure 3:**
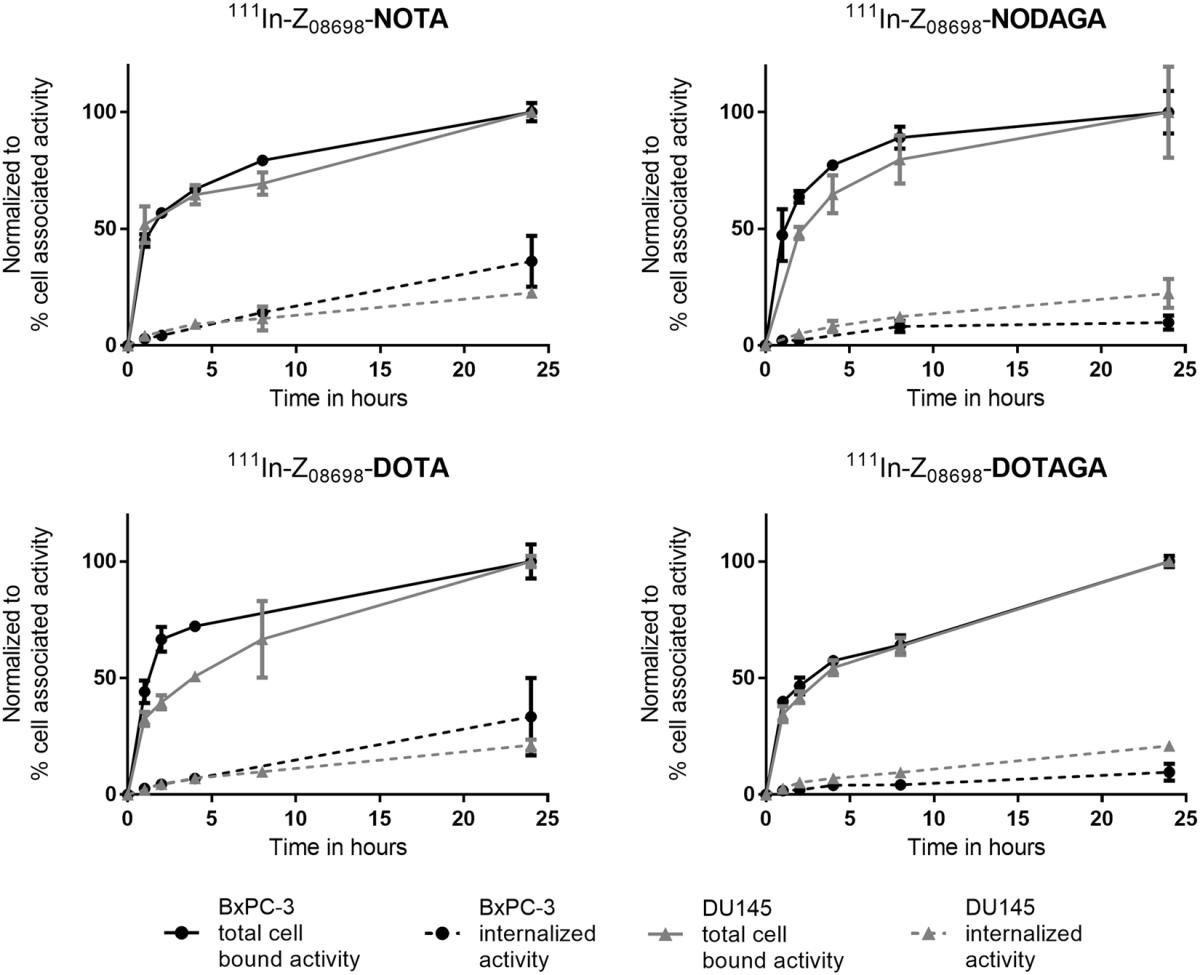
Cellular processing of ^111^In-Z_08698_-X on BxPC-3 (black) and DU145 cells (grey). Total cell bound activity (solid line) and internalized activity (dashed line) are displayed as percent of cell associated activity. The values were normalized to maximum cell-associated activity for each conjugate. Data are presented as an average ± standard deviation from three cell culture dishes.

Data regarding cellular processing are presented in Fig. 3. Cells were continuously incubated with 0.1 nM of the radiolabeled conjugates and, cell-membrane bound and internalized activity was analyzed at pre-determined timepoints. Overall, the association of all radiolabeled conjugates to the cells was rapid but the internalization rate was low. The internalized fraction in BxPC-3 cells was higher for ^111^In-Z_08698_-NOTA and ^111^In-Z_08698_-DOTA compared to ^111^In-Z_08698_-NODAGA and ^111^In-Z_08698_-DOTAGA. After 24 h continuous incubation, the internalized portion of the activity was over 30% of cell-associated activity for the NOTA- and DOTA-conjugates and around 10% for the NODAGA- and DOTAGA-conjugates for BxPC-3 cells. In DU145 cells, internalization was around 20% of cell-associated activity at 24 h without any significant difference between the conjugates.

### Real-time kinetics of binding to HER3 expressing BxPC-3 cells

Association rates (k_a_), dissociation rates (k_d_) and equilibrium dissociation constants (K_D_) were analyzed on living cells in real-time using LigandTracer yellow (Ridgeview Instruments AB). Results are shown in Table 3. For all conjugates, K_D_-values were in the picomolar range. ^111^In-Z_08698_-DOTAGA showed the highest affinity and ^111^In-Z_08698_-NOTA showed the lowest affinity in this assay.

**Table 3:** *In vitro* binding kinetics.

	^111^In-Z_08698_-NOTA	^111^In-Z_08698_-NODAGA	^111^In-Z_08698_-DOTA	^111^In-Z_08698_-DOTAGA
k_a_ (1/M*s)	10 × 10^4^ ± 2 × 10^4^	1.7 × 10^5^ ± 0.8 × 10^5^	1.5 × 10^5^ ± 0.4 × 10^5^	1.3 × 10^5^ ± 0.8 × 10^5^
k_d_ (1/s)	1.4 × 10^−5^ ± 0.7 × 10^−5^	2 × 10^−5^ ± 3 × 10^−5^	1.3 × 10^−5^ ± 0.7 × 10^−5^	0.23 × 10^−5^ ± 0.08 × 10^−5^
K_D_ (pM)	160 ± 50	21 ± 7	80 ± 50	8 ± 6

Equilibrium dissociation constant (K_D_), association rate (k_a_) and dissociation rate (k_d_) of ^111^In-Z_08698_-X. k_a_, k_d_ and K_D_ were measured in real time on BxPC-3 cells at room temperature.

### *In vivo* biodistribution

Biodistribution was studied in Balb/c nu/nu mice bearing BxPC-3 xenografts. Mice were injected with 2 µg (30 kBq) of ^111^In-Z_08698_-X and sacrificed 4 h and 24 h pi to collect tumors and tissues. The results are presented in Tables 4 and 5. The general biodistribution pattern was characteristic for affibody molecules (fast blood clearance and renal excretion) and for anti-HER3-affibody molecules in particular (elevated uptake in lung, liver, stomach, salivary glands and intestines).

**Table 4:** Activity Uptake *in vivo*.

	^111^In-Z_08698_-NOTA		^111^In-Z_08698_-NODAGA		^111^In-Z_08698_-DOTA		^111^In-Z_08698_-DOTAGA	
	4 h	24 h	4 h	24 h	4 h	24 h	4 h	24 h
Blood	0.79 ± 0.09^a,b,c^	0.32 ± 0.02^a,b,c^	0.51 ± 0.06^a,d^	0.17 ± 0.02^a^	0.63 ± 0.05^b,d,f^	0.167 ± 0.008^b^	0.49 ± 0.05^c,f^	0.19 ± 0.02^c^
Sal. Gland	2.4 ± 0.3^c^	1.85 ± 0.06^b^	2.5 ± 0.1^d,e^	1.7 ± 0.2	2.1 ± 0.2^d,f^	1.6 ± 0.2^b^	1.82 ± 0.03^c,e,f^	1.7 ± 0.2
Lung	2.4 ± 0.6	1.13 ± 0.06^a,b,c^	1.8 ± 0.2	0.9 ± 0.1^a^	1.82 ± 0.07	0.9 ± 0.1^b^	1.7 ± 0.1	0.88 ± 0.05^c^
Liver	10 ± 1^b,c^	4.6 ± 0.2^a,b,c^	7 ± 2^e^	3.6 ± 0.4^a^	6.2 ± 0.6^b,f^	3.5 ± 0.5^b^	5.0 ± 0.3^c,e,f^	3.4 ± 0.3^c^
Spleen	1.1 ± 0.1^a,b,c^	0.93 ± 0.08	0.88 ± 0.10^a,e^	0.9 ± 0.2	0.847 ± 0.009^b,f^	0.9 ± 0.2	0.562 ± 0.007^c,e,f^	0.7 ± 0.1
Stomach	2.6 ± 0.3^a,b,c^	1.5 ± 0.4	2.1 ± 0.2^a,e^	1.2 ± 0.3	1.8 ± 0.1^b^	1.2 ± 0.3	1.6 ± 0.2^c,e^	1.4 ± 0.3
Small intestine	7 ± 3	2.8 ± 0.8	4.7 ± 0.9	3.0 ± 0.4	6.5 ± 1.0^f^	2.6 ± 0.8	3.3 ± 0.3^f^	2.6 ± 0.5
Kidney	240 ± 15	181 ± 35	260 ± 29^d^	224 ± 18^d^	216 ± 19^d^	176 ± 19^d,f^	224 ± 21	231 ± 40^f^
Tumor	4.1 ± 0.7	3.2 ± 0.2	4 ± 1	3.2 ± 0.3	3.4 ± 0.7	2.8 ± 0.5	3.2 ± 0.5	3.4 ± 0.5
Muscle	0.34 ± 0.05^a,c^	0.24 ± 0.02^a,c^	0.22 ± 0.02^a^	0.209 ± 0.008^a,e^	0.28 ± 0.08	0.24 ± 0.03 ^f^	0.21 ± 0.05^c^	0.17 ± 0.03^c,e,f^
Bone	0.7 ± 0.1^a,b,c^	0.44 ± 0.03	0.50 ± 0.04^a,e^	0.4 ± 0.1	0.46 ± 0.04^b,f^	0.5 ± 0.2	0.30 ± 0.04^c,e,f^	0.4 ± 0.1
GI tract	7.4 ± 0.3	5.5 ± 0.5	9 ± 1	5.4 ± 0.5	6 ± 2	5 ± 1	6.5 ± 0.7	4.9 ± 0.8
Carcass	14 ± 1	10.1 ± 0.6	11.8 ± 0.5	8.4 ± 0.9	11.5 ± 0.6	8.5 ± 0.9	10 ± 1	9.1 ± 0.3

*In vivo* biodistribution of ^111^In-Z_08698_-X 4 hours and 24 hours pi in female Balb/c nu/nu mice bearing BxPC-3 xenografts. Uptake is presented as percent of injected dose per gram (%ID/g) and average of 3-4 animals per group. Data for GI tract and carcass is presented as %ID.

Significant difference (p < 0.05) between ^a 111^In-Z_08698_-NOTA and ^111^In-Z_08698_-NODAGA, ^b 111^In-Z_08698_-NOTA and ^111^In-Z_08698_-DOTA, ^c 111^In-Z_08698_-NOTA and ^111^In-Z_08698_-DOTAGA, ^d 111^In-Z_08698_-NODAGA and ^111^In-Z_08698_-DOTA, ^e 111^In-Z_08698_-NODAGA and ^111^In-Z_08698_-DOTAGA, ^f 111^In-Z_08698_-DOTA and ^111^ In-Z_08698_- DOTAGA.

**Table 5:** Tumor-to-Organ ratios.

	^111^In-Z_08698_-NOTA		^111^In-Z_08698_-NODAGA		^111^In-Z_08698_- DOTA		^111^In-Z_08698_-DOTAGA	
	4 h	24 h	4 h	24 h	4 h	24 h	4 h	24 h
Blood	5.1 ± 0.6	10.0 ± 0.4^a,b,c^	7 ± 2	19 ± 3^a^	5 ± 1	17 ± 3^b^	6.0 ± 0.9	18 ± 3^c^
Sal. Gland	1.7 ± 0.2	1.74 ± 0.08	1.4 ± 0.4	1.9 ± 0.1	1.7 ± 0.3	1.8 ± 0.2	1.62 ± 0.07	2.0 ± 0.2
Lung	1.7 ± 0.2	2.9 ± 0.3^c^	1.9 ± 0.5	3.5 ± 0.7	1.9 ± 0.4	3.3 ± 0.3	1.7 ± 0.2	3.9 ± 0.4^c^
Liver	0.43 ± 0.04^c^	0.70 ± 0.06^a,c^	0.5 ± 0.2	0.9 ± 0.1^a^	0.6 ± 0.1	0.8 ± 0.2	0.61 ± 0.06^c^	1.0 ± 0.2^c^
Spleen	3.6 ± 0.2^c^	3.5 ± 0.3^c^	3.9 ± 1.0^e^	3.6 ± 0.8	4 ± 0.8^f^	3.0 ± 0.3^f^	5.3 ± 0.3^c,e,f^	4.7 ± 0.6^c,f^
Stomach	1.6 ± 0.2^c^	2.4 ± 0.9	1.7 ± 0.5	2.7 ± 0.5	1.9 ± 0.4	2.4 ± 0.4	2.0 ± 0.1^c^	2.5 ± 0.6
Small intestine	0.6 ± 0.1^c^	1.2 ± 0.3	0.6 ± 0.2	1.05 ± 0.09^e^	0.52 ± 0.05^f^	1.2 ± 0.5	0.89 ± 0.02^c,f^	1.3 ± 0.2^e^
Muscle	12 ± 2^c^	14 ± 2^c^	16 ± 5	15 ± 1	12 ± 2	12 ± 3^f^	15 ± 2^c^	21 ± 5^c,f^
Bone	5.8 ± 0.7^c^	7.3 ± 0.5	7 ± 2	8 ± 2	8 ± 2	6 ± 1	11 ± 3^c^	9 ± 3

*In vivo* biodistribution of ^111^In-Z_08698_-X 4 hours and 24 hours pi in female Balb/c nu/nu mice bearing BxPC-3 xenografts. Tumor-to-organ ratios are presented as average of 3–4 animals per group.

Significant difference (p < 0.05) between ^a 111^In-Z_08698_-NOTA and ^111^In-Z_08698_-NODAGA, ^b 111^In-Z_08698_-NOTA and ^111^In-Z_08698_-DOTA, ^c 111^In-Z_08698_-NOTA and ^111^In-Z_08698_-DOTAGA, ^d 111^In-Z_08698_-NODAGA and ^111^In-Z_08698_-DOTA, ^e 111^In-Z_08698_-NODAGA and ^111^In-Z_08698_-DOTAGA, ^f 111^In-Z_08698_-DOTA and ^111^In-Z_08698_- DOTAGA.

Tumor uptake 4 h pi was in the range of 3–4%ID/g and no significant difference in tumor uptake was observed between the tested conjugates. Retention of activity in tumors over time was high and no significant changes of activity uptake were observed between 4 and 24 h pi for any conjugate. Activity concentration in blood was highest for ^111^In-Z_08698_-NOTA and it was significantly higher than the concentration of the other conjugates at both time points.

All conjugates demonstrated elevated initial uptake in organs with endogenous expression of mErbB3 (salivary gland, lung, stomach, intestines and especially liver). Hepatic uptake of ^111^In-Z_08698_-DOTA and ^111^In-Z_08698_-DOTAGA was significantly lower than for ^111^In-Z_08698_-NOTA. The liver uptake of ^111^In-Z_08698_-DOTAGA was the lowest at both studied time points. The difference was most pronounced at 4 h pi where uptake of the DOTAGA-conjugate was nearly two-fold lower than for the NOTA-conjugate, which demonstrated the highest hepatic uptake. ^111^In-Z_08698_-NOTA had also the highest uptake in lungs, stomach, spleen, muscle and bone at 4 h pi. Significant reduction of activity accumulation was observed in liver, lung and blood for all conjugates over time. Still, ^111^In-Z_08698_-NOTA demonstrated significantly higher uptake in liver, blood and lung than all other conjugates at 24 h.

The decrease of activity uptake in normal tissue together with good retention in tumors manifested in an increase of tumor-to-organ ratios between 4 and 24 h pi for the majority of the studied tissues, except spleen. Tumor-to-organ ratios did not differ significantly between ^111^In-Z_08698_-NODAGA, ^111^In-Z_08698_-DOTA and ^111^In-Z_08698_-DOTAGA. However, at 24 h pi ^111^In-Z_08698_-NOTA presented almost two-fold lower tumor-to-blood-ratio than the other conjugates. ^111^In-Z_08698_-DOTAGA was the only conjugate providing significantly better tumor-to-organ ratios than ^111^In-Z_08698_-NOTA for liver, spleen and muscle at both time points.

### SPECT-Imaging

**Figure 4:**
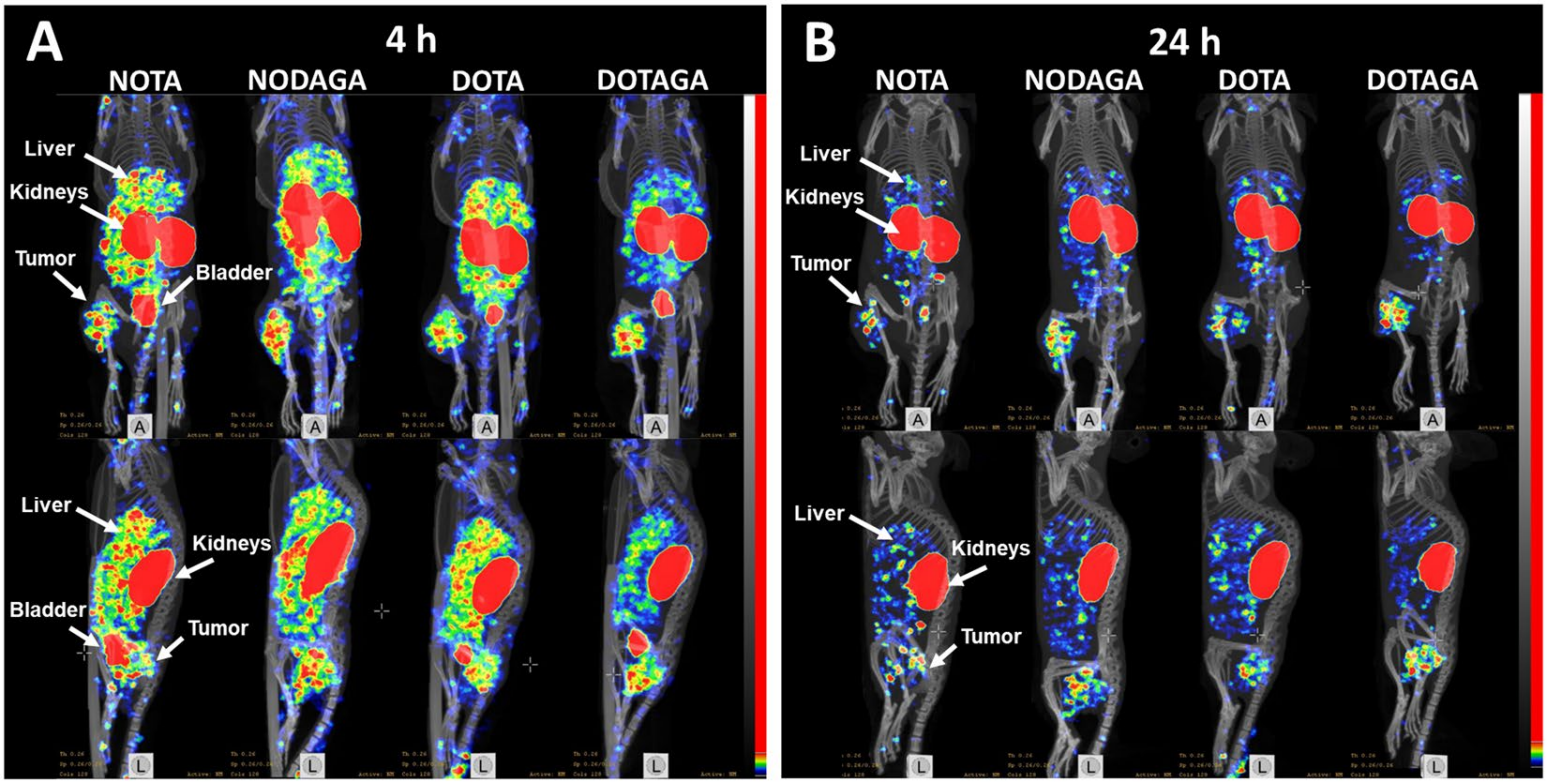
SPECT-CT imaging of ^111^In-Z_08698_-X. MIP images coronal and sagittal view at A) 4 h pi and B) 24 h pi. Mice were bearing BxPC-3 xenografts and injected with 2 µg (~1.5 MBq) ^111^In-Z_08698_-X.

SPECT-CT imaging of ^111^In-Z_08698_-X confirmed the results of the biodistribution and images are displayed in Fig. 4. HER3-expressing BxPC-3 xenografts could be clearly visualized. Uptake related to natural expression of mErbB3 could be observed in salivary glands, GI-tract and liver. ^111^In-Z_08698_-NOTA had the highest activity accumulation in liver at both timepoints.

## Discussion

The development of therapeutic agents against HER3 urges simultaneous development of reliable methods for patient stratification. Radionuclide-based molecular imaging provides a non-invasive and repeatable method to identify patients who might benefit from targeted therapy^17,18,30^. Radiolabeled monoclonal antibodies against HER3 have recently demonstrated the capacity to image overexpression and evaluate HER3 receptor occupancy in patients with HER3-positive tumor lesions^31,32^. However, the rather large size (150 kDa) and the slow *in vivo* kinetics of monoclonal antibodies result in a moderate imaging contrast^33^. In addition, unspecific accumulation in tumors due to the enhanced permeability and retention effect reduces sensitivity and specificity of monoclonal antibodies as imaging probes^34^. The use of affibody molecules for imaging of HER3 expression could provide a promising alternative.

Affibody molecules are small affinity proteins (58 amino acids) and even small modifications of these targeting molecules can have significant impact on their biodistribution and tumor-to-non-tumor ratios which could be a useful instrument to optimize their imaging properties. The only difference between the affibody molecules used in this study was the chelator conjugated to the C-terminus. Earlier data have shown that even such small differences might cause noticeable modification in biodistribution of radiolabeled affibody molecules^26,27^.

Previous studies demonstrated that there are two characteristics of the radiometal-chelator complex having major impact on the behavior of anti-HER2 affibody molecules: complex charge and geometry^26–28^. The charge of the radiometal/chelator complex can vary depending on the valency of the metal and the number of available charged groups of the chelator^35^. The geometry of the formed complex is influenced by the size of the radiometal, which may require co-ligands for stable chelation^35–37^. Indium is a trivalent metal and its complexes with NOTA and DOTA are well studied^35,37^. Indium is hexa-coordinated with NOTA in a distorted prismatic structure and octa-coordinated with DOTA resulting in a square antiprism-like structure^37,38^. Their respective derivatives NODAGA and DOTAGA are assumed to form similar complexes^37^. Hexa-coordination of indium with NOTA results in an overall positively charged complex (Fig. 1). ^111^In-NODAGA and ^111^In-DOTA complexes are neutral in charge, but differ in size and structure. Lastly, the DOTAGA chelator carries an additional carboxylic arm resulting in a negatively charged complex. While the charge and geometry of radiometal complexes are important per se, their interplay with amino acids in the binding site is also essential. The binding site of affibody scaffold contains 13 amino acids of 58 (22%), and physicochemical properties of side chains of binding amino acids may strongly influence off-target interactions. Modifications of binding sites of anti-HER2 and anti-HER3 affibody molecules had significant influence on blood clearance rate and hepatic uptake, even when the same labelling approach was used^22,39^. Therefore it was necessary to evaluate if observations made for anti-HER2 affibody molecules are still valid for anti-HER3 counterparts.

Despite of differences in size and denticity of radiometal-chelator complexes all conjugates were stably radiolabeled with indium-111. However, labeling yields were much higher for the NODAGA and DOTAGA-conjugated variants. Difference in labeling yields did not correlate with size of the chelator (triaza vs. tetraaza), chelator’s denticity or presence of impurities. Impurities were observed in the ESI-MS spectra of NOTA and NODAGA, possibly due to chelation of iron and copper. It could be speculated that the accessibility of the chelator was influenced by the length of the linker between the affibody molecule and the chelator. Nevertheless, such steric hindrance did not affect the targeting properties of the conjugates. All radiolabeled conjugates bound to the target with high affinity. When comparing the binding affinity of ^111^In-Z_08698_-NOTA with its previously investigated (HE)_3_-containing counterpart, ^111^In-(HE)_3_-Z_08698_-NOTA had a considerably better affinity (5.4 ± 0.4 pM^20^). Thus, absence of the hydrophilic (HE)_3_-tag on the N-terminus might have an effect on the binding affinity.

Analysis of cellular processing demonstrated that the overall internalization rate was slow, even though ^111^In-Z_08698_-NOTA and ^111^In-Z_08698_-DOTA showed somewhat higher internalization rate in BxPC-3 cells compared to the other conjugates. However, no differences in internalization pattern were seen between conjugates in DU145 cells. Since members of the human epidermal growth factor receptor family act through dimerization, cellular processing and internalization rate can be influenced by varying expression levels of the target receptor and potential presence of dimerization partners. This is particularly important in the case of HER3 which is dependent on hetero-dimerization with other HER-family members for signaling, i.e. HER2 and EGFR^1^. Hence, different levels of HER co-expression can explain small observed differences between cell lines. In addition, it has been previously suggested that the radiometal-chelator complex can influence the cellular processing of affibody molecules^26,27^.

The overall *in vivo* distribution patterns of the tested conjugates were comparable with the previously studied anti-HER3 affibody molecule (HE)_3_-Z_08698_-NOTA labeled with indium-111, gallium-68 and radiocobalt^20,21,29^. Tumor uptake at 4 h pi was in the range of 3–4%ID/g and the retention of activity in tumors was good, therefore it can be concluded that the choice of chelator had no significant influence on tumor uptake. Despite 20-fold difference in affinity the initial tumor uptake was similar. This could reflect that other factors such as tumor size, shape or vascularization have an effect on tumor uptake. Nevertheless, ^111^In-Z_08698_-DOTAGA conjugates had the best tumor retention, which correlated with the best affinity found for this conjugate. Blood clearance of ^111^In-Z_08698_-NOTA was significantly slower than for the other conjugates. In addition, its concentration in blood was notably higher than for the previously studied (HE)_3_-containing variants (0.1–0.27%ID/g)^20,21,29^. The relatively slow blood clearance of ^111^In-Z_08698_-NOTA might be a result of weak interaction with blood proteins, which was previously balanced by the presence of the hydrophilic (HE)_3_-tag^20^. Overall, it can be concluded that the choice of the radiometal-chelator complex influenced blood clearance, affinity and tumor retention.

**Figure 5:**
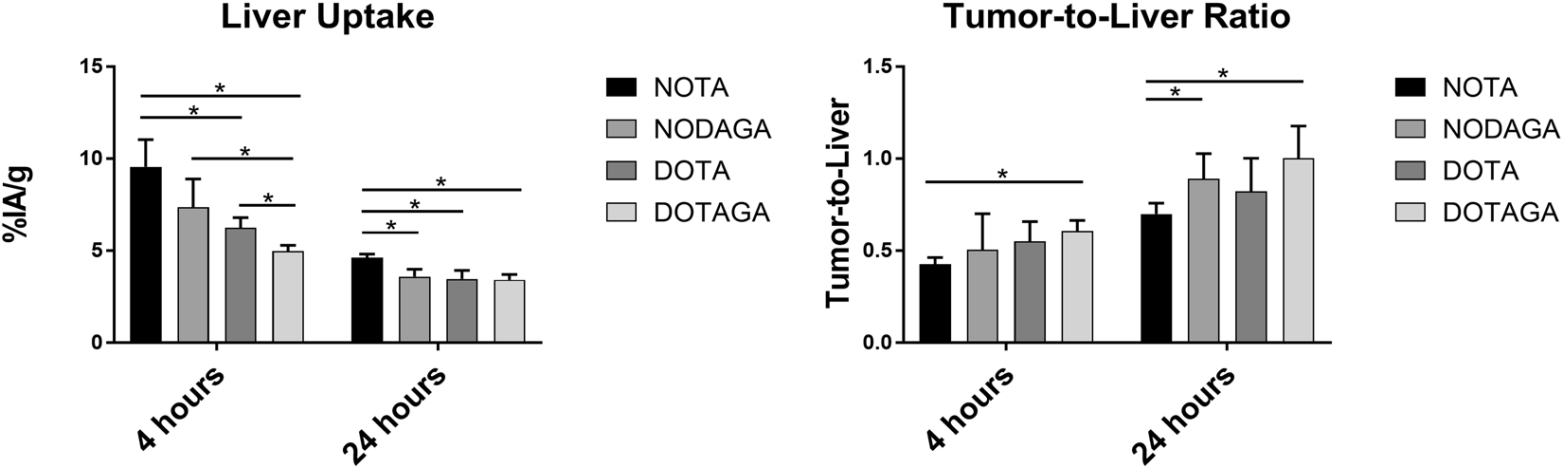
Activity uptake in liver (left) and tumor-to-liver ratios for all ^111^In-Z_08698_-X conjugates over time studied in female Balb/c nu/nu mice bearing BxPC-3 xenografts. Significant differences between conjugates are marked with asterisks.

Even though the choice of chelator did not affect the tumor uptake, the results of the current study suggest that the radiometal-chelator complex greatly influences the uptake in organs with endogenous expression of HER3, particularly in liver, both in initial uptake (4 h pi) and retention (24 h pi). It was hypothesized earlier that hepatic uptake of affibody molecules is mediated by two mechanisms: a primary receptor specific mechanism and a secondary off-target mechanism possibly related to lipophilicity and charged surface groups of the affibody scaffold^29,40^. Previous reports have also shown that the increase of negative charge on the N- or C-terminus significantly reduces the hepatic uptake of affibody molecules^27,28^. This was in agreement with the observation that the uncharged cobalt-NOTA complex provided lower hepatic uptake than positively charged indium- and gallium-NOTA complexes when conjugated to (HE)_3_-Z_08698_^20,21,29^. The results of the current study support this hypothesis. ^111^In-Z_08698_-DOTAGA carrying a negative charge at the C-terminus had the lowest uptake in liver and significantly lower compared with the ^111^In-NOTA conjugate. Figure 5 illustrates the differences in hepatic uptake and tumor-to-liver ratio at 4 and 24 h pi.

^111^In-Z_08698_-NODAGA and ^111^In-Z_08698_-DOTA have neutral charge at the C-terminus and both showed lower uptake in liver than ^111^In-Z_08698_-NOTA, but their hepatic uptake did not differ significantly from each other. ^111^In-Z_08698_-DOTAGA also showed the lowest uptake in organs with endogenous expression of mErbB3 at 4 h pi, which may be related to a reduced bioavailability of the conjugate due to rapid blood clearance. As a result, ^111^In-Z_08698_-DOTAGA is considered the most favorable variant among the studied conjugates.

The differences in uptake between conjugates also reflected on the tumor-to-organ ratios. Tumor-to-organ-ratios for ^111^In-Z_08698_-DOTAGA were significantly higher than ^111^In-Z_08698_-NOTA for liver, spleen, stomach, muscle and bone at 4 and 24 h pi. Hence, introduction of a negative charge at the C-terminus of the anti-HER3 affibody molecules improved the contrast in these organs. This was furthermore supported by the SPECT-CT images (Fig. 4). At both time points, the xenografts could be clearly visualized, but the best imaging contrast was provided by ^111^In-Z_08698_-DOTAGA 24 h pi.

## Conclusions

The biodistribution of anti-HER3 affibody molecules is influenced by the combination of chelator and radiometal. It can be concluded that an increased negative charge at the C-terminus of anti-HER3 affibody molecules decreases the activity uptake in liver by reducing unspecific uptake. SPECT-CT imaging of ^111^In-Z_08698_-X (X = NOTA, NODAGA, DOTA, DOTAGA) confirmed the results of the biodistribution. Thus, the ^111^In-Z_08698_-DOTAGA conjugate is considered superior to the other variants for imaging of HER3 overexpression *in vivo*.

## Material and Methods

All data generated or analysed during this study are included in this published article (and its Supplementary Information files) and are available from the corresponding author on reasonable request.

HER3 expressing cell lines, BxPC-3 (pancreatic cancer) and DU145 (prostate cancer), were purchased from American Type Tissue Culture Collection (ATTC via LGC Promochem, Borås, Sweden) and cultured in RMPI-1640 media supplemented with 1% penicillin and 1% L-glutamine (all from Biochrom, Berlin, Germany) and 10% fetal bovine serum (Sigma-Aldrich, Germany) at 37 °C with 5% CO_2_. Trypsin-EDTA solution (0.25% trypsin, 0.02% EDTA in buffer) was used to detach cells.

Indium-111 was purchased in form of ^111^In-chloride from Mallinckrodt Pharmaceuticals (Staines-upon-Thames, United Kingdom). Radioactivity content of samples in *in vitro* and *in vivo* experiments was measured with a 3-inch NaI(Tl) detector (1480 Wizard; Wallac Oy, Turku, Finland). All animal studies were performed in accordance with the national legislation on protection of laboratory animals and approved by the Ethics Committee for Animal Research in Uppsala, Sweden.

Results are presented as average with standard deviation. Statistical significance (p < 0.05) was evaluated by unpaired, two-tailed t-test using GraphPad Prism (version 7.03 for Windows, GraphPad Software, San Diego, CA, USA).

### Production, conjugation and purification

The HER3-binding affibody Z_HER3:08698_ was produced and purified according to similar procedures as described previously^20^.

The Z_08698_ was produced in *E*. *coli* BL21*(DE3) (Thermo Fisher Scientific) in an overnight culture at 25 °C after induced expression with 100 μM IPTG (Isopropyl β-D-1-thiogalactopyranoside) at an OD_600_ of 0.8. Following cell lysis with French press, the supernatant was heated to 90 °C for 10 min with subsequent incubation on ice for 20 min, followed by centrifugation to remove precipitated proteins. The affibody molecule was purified on an ÄKTAexplorer (GE Healthcare, Uppsala, Sweden) using a 1 ml Resource S cation exchange column (GE Healthcare), running in 20 mM mM MES (2-(N-morpholino)ethanesulfonic acid, pH 6) and eluted by 20 mM MES with 1 M NaCl (pH 6). The buffer of the eluate was changed to 20 mM NH_4_Ac (pH 5.5) and the proteins were freeze-dried.

The proteins were dissolved in 20 mM NH_4_Ac (pH 5.5) and reduced with a molar concentration of tris(2-carboxyethyl)phosphine (TCEP) equal to the protein concentration for 30 min at 37 °C. The proteins were incubated at 37 °C for 90 min with ten-fold molar excess of maleimide derivatives of DOTA, DOTAGA, NOTA and NODAGA (CheMatech) for site-specific conjugation to the C-terminal cysteine on the affibody. Metal ion contaminations were removed from all buffers with Chelex 100 resin (Bio-Rad Laboratories).

After the site-specific conjugation, reverse-phase high performance liquid chromatography (RP-HPLC) on a 1200 series HPLC system using a Zorbax 300SB-C18 semi-preparative column (Agilent Technologies, Santa Clara, CA) was used for purification. Water with 0.1% trifluoroacetic acid was used as running buffer and an acetonitrile gradient was used for elution.

### Characterization

The purity of the four conjugates was determined using RP-HPLC and an analytical Zorbax 300SB-C18 column (Agilent Technologies) with a 20–40% acetonitrile elution gradient over 20 min with a flow rate of 1 ml/min.

Circular dichroism spectroscopy was performed using a Chirascan spectropolarimeter (Applied Photophysics, United Kingdom) with an optical path length of 1 mm, to analyze the alpha-helical content, thermal stability and refolding capacity of the four conjugates at a concentration of 0.25 mg/ml. The thermal stability was evaluated by measuring the change in ellipticity at 221 nm during heating (5 °C/min) from 20 to 90 °C. The melting temperatures (T_m_) were approximated from the data acquired from variable temperature measurements (VTM) by curve fitting using a Boltzmann Sigmoidal model (GraphPad Prism, version 7). The refolding capacity was assessed by comparing spectra obtained from measurements at wavelengths in the range 195–260 nm at 20 °C, before and after thermal denaturation.

ESI-MS with a 6520 Accurate-Mass Q-TOF LC/MS (Agilent Technologies) was used for confirmation of molecular masses of the purified conjugates.

The affinity of the conjugates to human HER3 was investigated using single-cycle kinetics on a BIAcore T200 system (GE Healthcare) using a CM5 sensor chip with immobilized His-hHER3 and mFc-hHER3 (Sino Biological) with immobilization levels of 1500 and 2500 resonance units (RU) respectively. Measurements were performed in duplicates. Five concentrations of each conjugate were sequentially injected in a single cycle with a contact time of 100 seconds for DOTA-, DOTAGA- and NODAGA-conjugated affibody and 50 seconds for NOTA-conjugated affibody, for each concentration. The acquired sensorgrams were analyzed using a Langmuir 1:1 kinetic model.

### Labeling of conjugates with indium-111

Conjugates (25 µg in 50 µl ammonium acetate buffer 0.2 M, pH 5.5) were incubated with 25 µl of ^111^In-chloride (20 MBq) for 40 minutes at 85 °C. For Z_08698_-DOTA incubation was prolonged to 60 minutes and performed in presence of acetonitrile. Radiolabeled conjugates were purified with disposable NAP5-columns (GE Healthcare) pre-equilibrated with 1% BSA/PBS and eluted with PBS. Labeling yield and radiochemical purity was analyzed by instant thin layered chromatography (ITLC). For analysis, 1 µl of radiolabeled solution was applied to silica gel-impregnated glass microfiber chromatography paper (Agilent Technologies, CA, USA), and samples were developed in citric acid (0.2 M). Radioactivity distribution was then measured with the Cyclone Storage Phosphor System and analyzed with OptiQuant image analysis software (Perkin Elmer, Waltham, MA, USA).

Stability of the radiolabeled conjugates was evaluated by incubating 2.5 µg of ^111^In-Z_08698_-X (here and further “radiolabeled affibody molecules”) at 37 °C with 100 µl human serum for 24 h. The samples were thereafter analyzed with ITLC.

### Real-time ligand-binding kinetics

The kinetics of radiolabeled affibody molecules binding to living BxPC-3 cells was measured in real time using LigandTracer yellow (Ridgeview Instruments AB, Vänge, Sweden) at room temperature, as previously described^41^. Concentrations of the radiolabeled affibody molecules were between 0.3 nM and 10 nM.

### *In vitro* studies

Binding specificity and cellular processing of radiolabeled conjugates were investigated using BxPC-3 and DU145 cells as described previously^20^. All experiments were performed in triplicates. Cells were seeded in 35 mm cell dishes one day prior to the experiment in a density of 10^6^ cells/dish (BxPC-3) or 0.7 × 10^6^ cells/dish (DU145).

For investigation of binding specificity, HER3 receptors were pre-saturated by addition of 50 nM unlabeled Z_08698_ to half of the dishes. After incubation for 15 min at room temperature, 0.1 nM of ^111^In-Z_08698_-X was added to all dishes and cells were incubated for 1 hour at 37 °C. Afterwards, detached cells were collected and radioactivity content was measured.

For cellular processing, cells were incubated continuously with 0.1 nM of ^111^In-Z_08698_-X at 37 °C. At pre-determined time points, a set of 3 dishes was analyzed. To collect the membrane bound radioactivity, cells were incubated with glycine buffer (0.2 M, pH 2.5, 4 M urea) on ice. In order to collect the internalized radioactivity, cells were further incubated at 37 °C with NaOH (1 M) for 30 min at 37 °C. Samples were measured in the automated gamma counter.

### *In vivo* biodistribution

Biodistribution of ^111^In-Z_08698_-X was investigated in female Balb/c nu/nu mice bearing BxPC-3 xenografts. To establish tumors, 5 × 10^6^ cells per mouse were implanted 17 days prior to the study. At the day of experiment, the average mouse weight was 18 ± 1 g and average tumor weight was 0.09 ± 0.05 g.

Mice were intravenously injected with 2 µg of ^111^In-Z_08698_-X (30 kBq) in 100 µl of 1% BSA/PBS. Non-labeled protein was used to adjust the amount of injected protein. Groups of 4 mice per data point were sacrificed 4 h and 24 h pi (pre-injected intra-peritoneal with Ketalar-Rompun solution, 10 mg/mL Ketalar and 1 mg/mL Rompun; 20 μL solution/gram of body weight). Blood samples were collected by heart puncture. Samples of lung, salivary glands, liver, stomach, small intestine, spleen, kidneys, muscle, bone and tumor were collected, weighed and measured for radioactivity content in the automated gamma counter. Radioactivity uptake was calculated as percent injected dose per gram (%ID/g). Results for carcass and gastrointestinal tract are expressed as %ID.

### Imaging

Whole body SPECT/CT scans of the mice injected with ^111^In-Z_08698_-X (2 µg, ~1.5 MBq) were performed at 4 and 24 h pi using nanoScan SPECT/CT (Mediso Medical Imaging Systems Ltd., Hungary). For the 4 h pi scan, the mice were placed under general anesthesia by administration of a mixture of Sevoflurane (3.5%), oxygen and medical air. CT was acquired at the following parameters: 50 keV energy peak, 670 μA, 480 projections, 5.26 min acquisition time. SPECT was carried out using ^111^In energy peaks of 245.4 keV and 171.3 keV, window width of 20%, matrix of 256 × 256, acquired for 1 h. CT raw files were reconstructed using Nucline 2.03 Software (Mediso Medical Imaging Systems, Hungary). SPECT raw data were reconstructed using Tera-Tomo™ 3D SPECT reconstruction technology. Images are presented as maximum intensity projection (MIP) in RGB (red, green and blue) color scale.

## Electronic supplementary material

Supplimentary information
