# Supplementary material for: Optimization of HER3 expression imaging using affibody molecules: Influence of chelator for labeling with indium-111
Source: Sci Rep. 2019 Jan 24;9:655. doi: 10.1038/s41598-018-36827-w (PMC6345776; doi:10.1038/s41598-018-36827-w)
Supplement: Supplementary file 1 — Supplimentary information [file 41598_2018_36827_MOESM1_ESM.pdf]

Rinne SS, Dahlsson Leitao C, Mitran B, Bass TZ, Andersson KG, Tolmachev V, Ståhl S, Löfblom J, Orlova A

Optimization of HER3 expression imaging using affibody molecules: Influence of chelator for labeling with indium-111

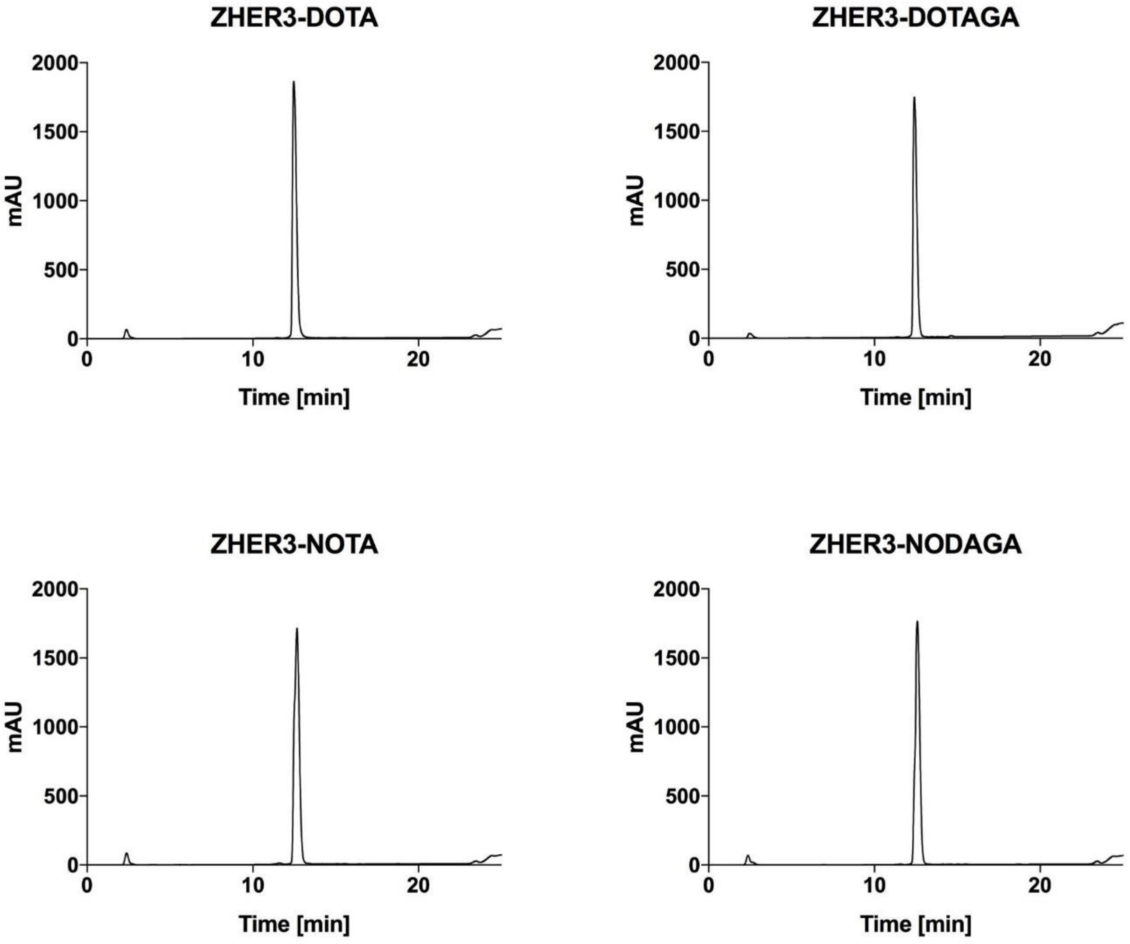

Figure S1: Purity determination. The purity of the four conjugates was evaluated by absorbance measurement at 220 nm using RP-HPLC.

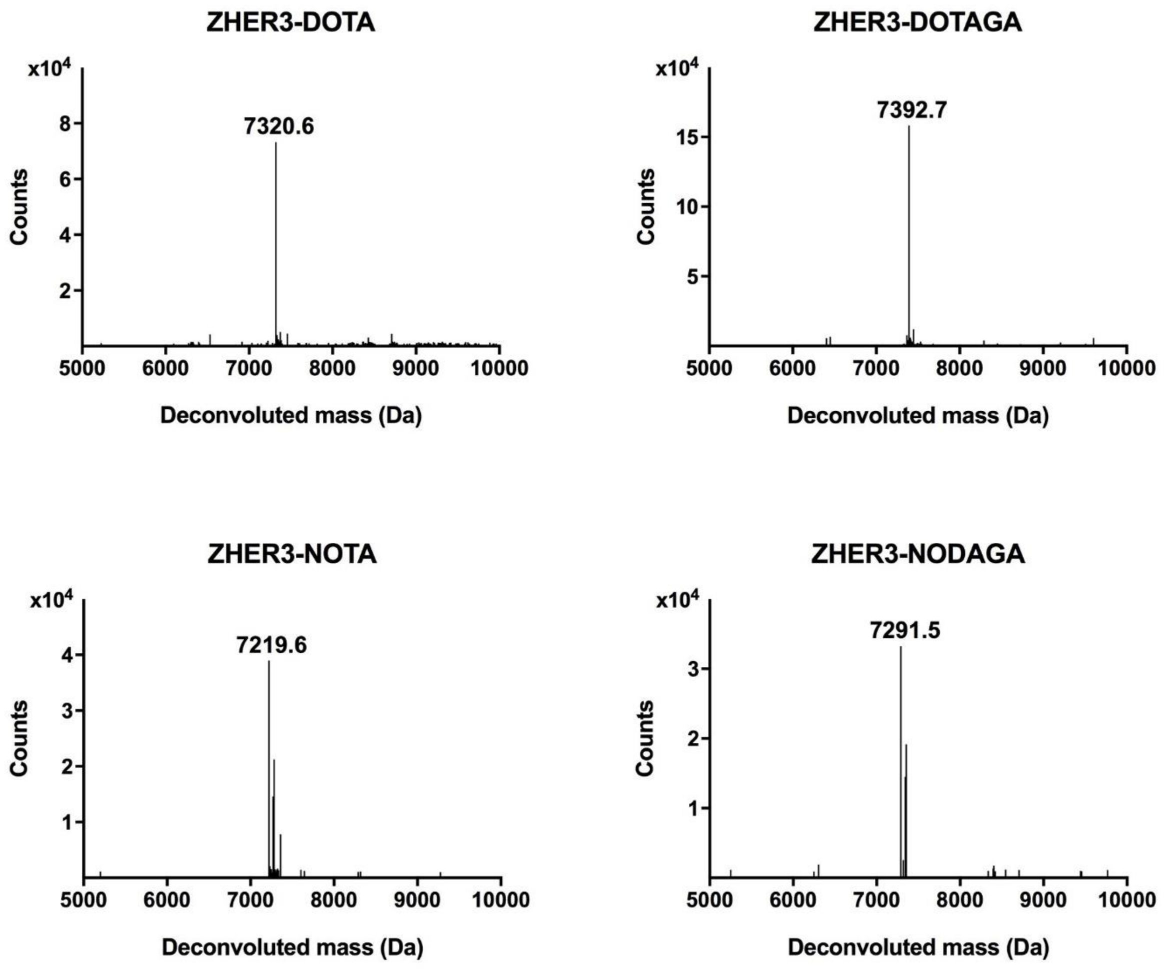

Figure S2: Mass determination. Results from ESI-MS confirming the identity of the conjugates. The experimental molecular masses of the observed peaks are in accordance with the theoretical masses shown in Table 1.

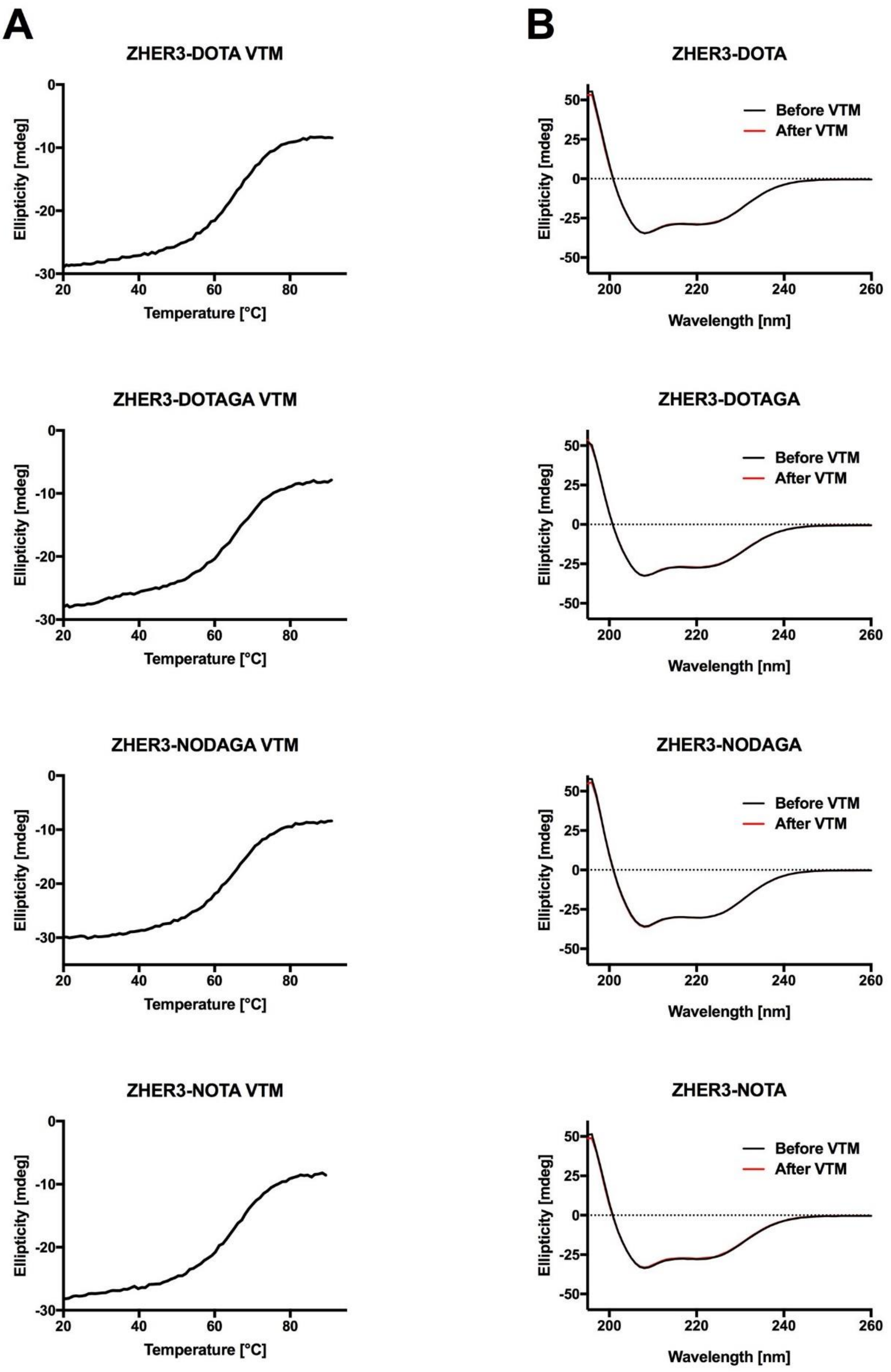

Figure S3: Analysis of thermal stability and refolding capacity of conjugates. A) Variable temperature measurement (VTM) spectra obtained at 221 nm while heating the sample from 20°C to 90°C. B) overlay of circular dichroism spectra (195-260 nm) before and after thermal denaturation. Melting temperatures (T_m) were determined by fitting the curves using a Boltzmann Sigmoidal model. The determined T_m values for the four conjugates are presented in Table 1.

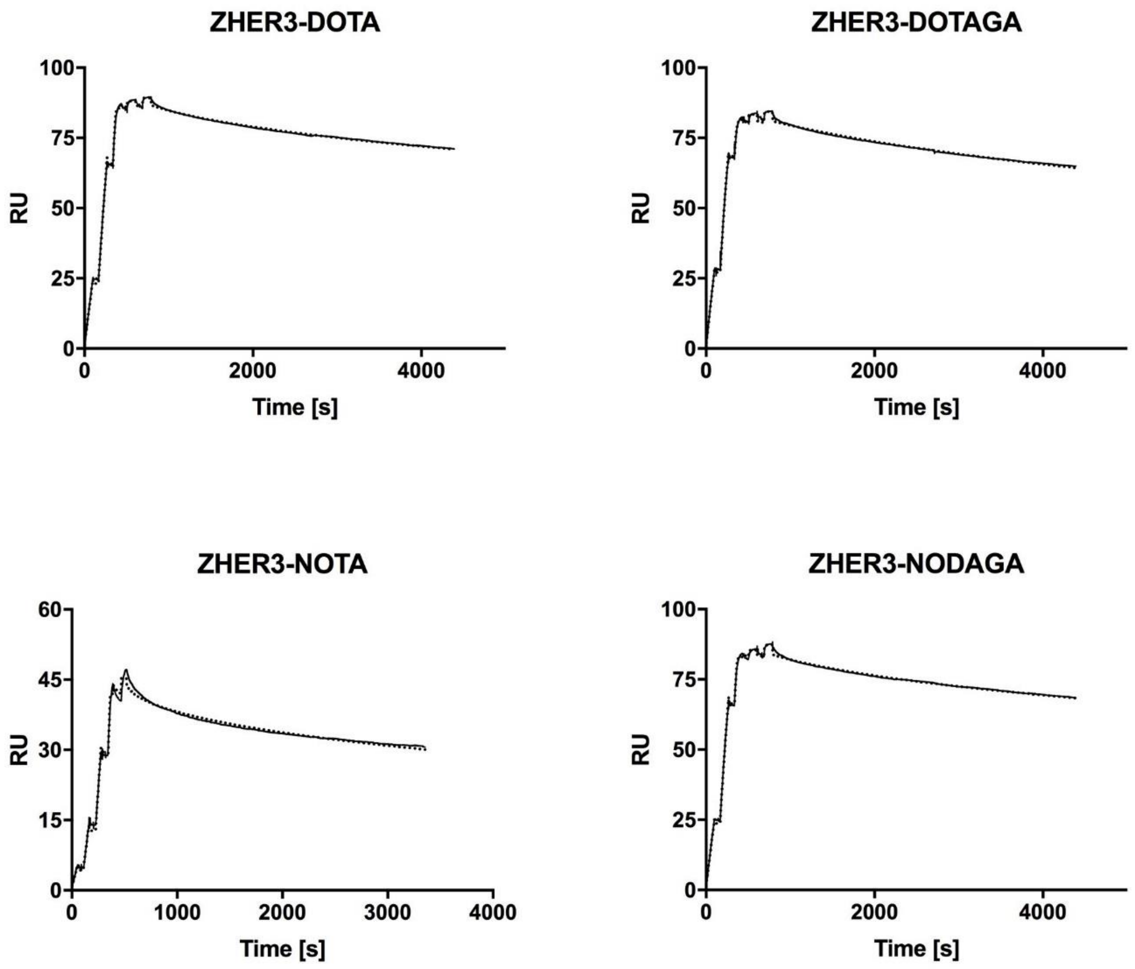

Figure S1: Representative experimental sensorgrams (solid) with fitted curves (dashed) from SPR analysis. Immobilized human HER3 was subjected to five concentrations (3.125, 6.25, 12.5, 25 and 50 nM) of each conjugate in a single cycle. Monovalent affinities, based on a Langmuir 1:1 model, are presented in Table 1.
